# Carbapenem resistance in the Dominican Republic: clinical characteristics, genotypic profiles, and risk factors

**DOI:** 10.1017/ash.2024.503

**Published:** 2025-01-27

**Authors:** Ricardo Ernesto Hernández-Landa, Rita Rojas-Fermín, Anel Guzman-Marte, Katherine Peralta Agramonte, Missel María Jiménez Cedano, Surelly Alexandra Mora-Peralta, Patricia Sanrregré-Oven, Alfredo J. Mena Lora

**Affiliations:** 1 Hospital General Plaza de la Salud, Santo Domingo, Dominican Republic; 2 University of Illinois at Chicago, Chicago, IL, USA

## Abstract

**Objective:**

To characterize the clinical and microbiological features of carbapenem-resistant Enterobacterales (CRE) compared to carbapenem-susceptible Enterobacterales (CSE) in the Dominican Republic (DR), and to assess risk factors associated with CRE.

**Design:**

Retrospective case-control study.

**Setting:**

Hospital General Plaza de la Salud, a tertiary teaching hospital in Santo Domingo, DR, from January 2015 to June 2024.

**Patients:**

Patients with CRE infections were identified from microbiology records. For each year, a matched group of CSE cases was selected at a 2.5:1 ratio. A total of 101 CRE cases and 280 CSE cases were included.

**Methods:**

Data were collected on demographics, comorbidities, infection sources, hospital stay duration, antibiotic use, and microbiology results. Statistical analysis included univariate and multivariate logistic regression to identify independent risk factors for CRE.

**Results:**

CRE cases showed higher prevalence of Enterobacter (36.5%) and Klebsiella (38.5%), while Escherichia predominated in CSE (65.5%). CRE patients had longer hospital stays (mean 18.7 vs 4.6 days, *P* < 0.001), higher ICU admission rates (34.7% vs 3.6%, *P* < 0.001), and increased invasive procedure use (eg central venous catheters, 36.6% vs 5.4%, *P* < 0.001). Key risk factors included antibiotic use (OR 3.09, *P* < 0.001) and ICU stay (OR 3.60, *P*= 0.012). The peak CRE resistance rate was 3.47% in 2022, a 64% increase from pre-pandemic levels.

**Conclusions:**

CRE infections in the DR increased during the COVID-19 pandemic, associated with prolonged hospitalizations and critical care. Enhanced antimicrobial stewardship is essential to curb resistance.

## Background

Antimicrobial resistance (AMR) is a major global public health challenge.^1^ Unique factors present in low- and middle-income countries (LMICs) may contribute to AMR.^2^ Antimicrobials are inexpensive and easily accessible in many LMICs, often without prescriptions.^3–5^ This ease of access may have contributed to a rise in antimicrobial usage and AMR in LMICs in the past decade.^6^ Further increases in the use of antibiotics due to the COVID-19 pandemic threatens to worsen AMR.^7^

In the Dominican Republic (DR), studies have shown high levels of extended-spectrum beta-lactamase (ESBL) producing organisms in ambulatory patients. In this study, carbapenem resistance was not commonly seen in ambulatory patients.^8^ However, an increase in carbapenem resistance occurred in ambulatory settings between 2020 and 2021.^8^ There is a paucity of data on inpatient susceptibility patterns in the DR and the impact of the COVID-19 pandemic on antimicrobial resistance (AMR). Understanding local susceptibility patterns is key to help guide empiric therapy. We seek to describe resistance in the DR and the impact of the COVID-19 pandemic.

## Methods

### Study design

This is a retrospective case-control study assessing clinical and epidemiological characteristics of patients with carbapenem-resistant Enterobacterales (CRE) and carbapenem-susceptible Enterobacterales (CSE) infections from January 3, 2015 to June 27, 2024. The study was conducted at Hospital General Plaza de la Salud, a 289-bed tertiary teaching hospital located in Santo Domingo, DR. Patients with CRE infections were identified retrospectively from clinical microbiology records. For each year of the study period, a matched group of patients with CSE infections was randomly selected. The number of CSE cases chosen each year was matched at a ratio of 2.5 CSE for every CRE case identified that year, ensuring a comparable sample size between the two groups.

### Data collection

Data was extracted from electronic medical records, including demographics, comorbidities, source of infection, length of hospital stay, and antibiotic utilization patterns. Additionally, we reviewed microbiology results from clinical cultures to identify causal pathogens, assess antibiotic susceptibility profiles, confirm the presence of carbapenemase production, and performed genotype testing of CRE isolates.

### Microbiology

Bacterial identification and susceptibility testing were done in a Vitek 2 compact system (bioMérieux, France). Strains that were non-susceptible to at least one carbapenem (ertapenem, meropenem, imipenem) according to the Clinical and Laboratory Standards Institute (CLSI) breakpoints available for each year, were tested for carbapenemase production with the modified carbapenem inactivation method (mCIM, CLSI M100). Strains that tested positive were then confirmed and with a phenotype-based disc method (D73C MASTDiscs Combi Carba Plus, Mast Group LTD, Merseyside, UK), following the manufacturer indications. In 2023, the institution implemented a qualitative lateral flow immunoassay to detect and differentiate the five most prevalent carbapenemases families (NDM, IMP, VIM, OXA-48, and KPC; NG-test CARBA 5, Hardy Diagnostics, USA).

### Statistical analysis

Frequencies and means with standard deviations were calculated for qualitative and quantitative variables respectively to perform an univariable analysis. Odds ratios (ORs) were calculated to compare clinical and epidemiological characteristics between CRE and CSE cases and to identify variables associated with CRE infection. Variables demonstrating statistically significant associations (*P* < 0.05 and a valid CI interval) in the bivariable analysis were subsequently incorporated into a multivariable logistic regression model. This analysis was conducted to identify independent risk factors for CRE infection while controlling for potential confounding variables. Statistical analyses were performed using JASP statistical analysis software.

### Ethics

The institutional review board at the Hospital General Plaza de la Salud reviewed and approved this study.

## Results

**Figure 1.**
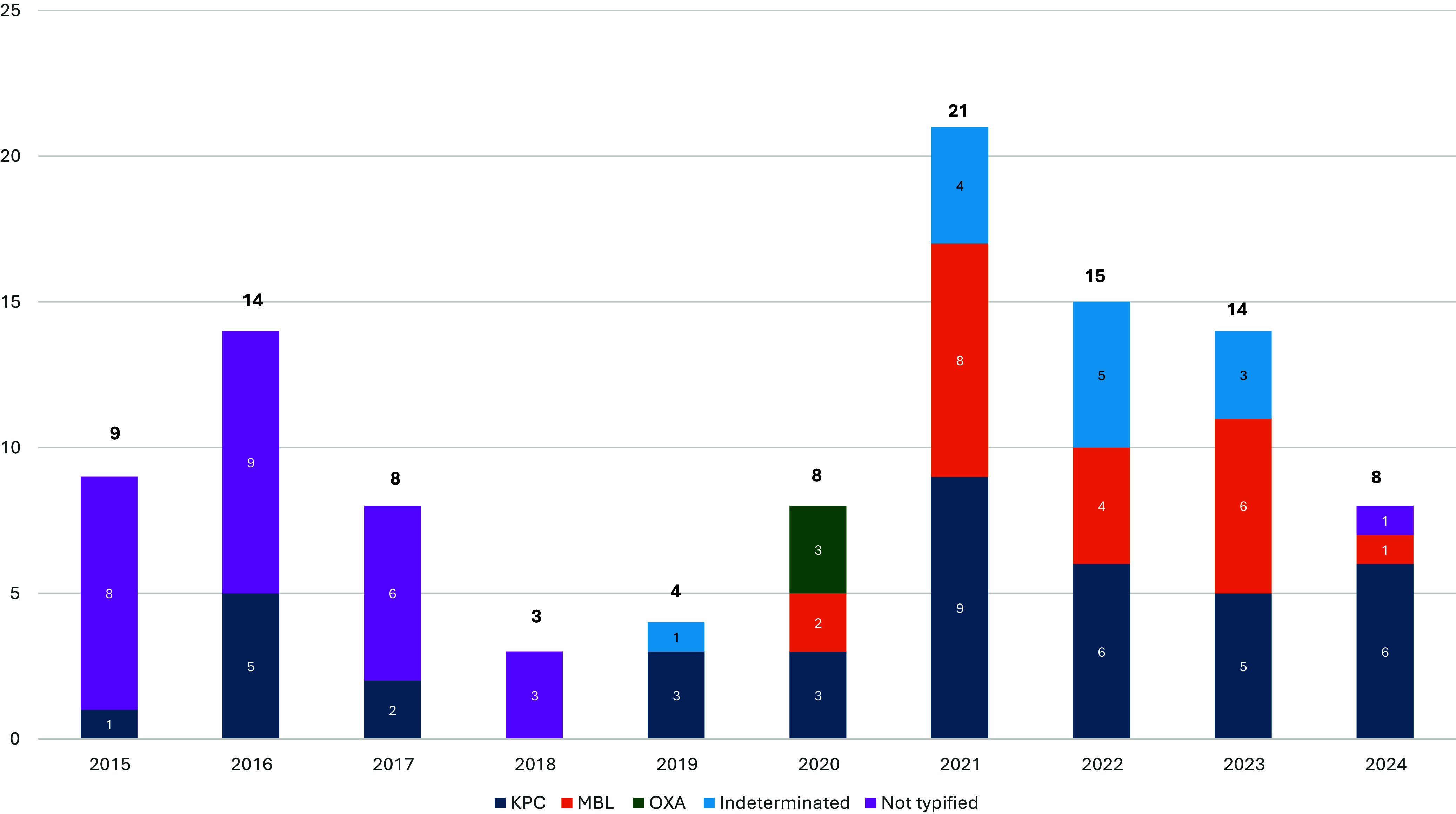
Carbapenem resistance by year.

**Figure 2.**
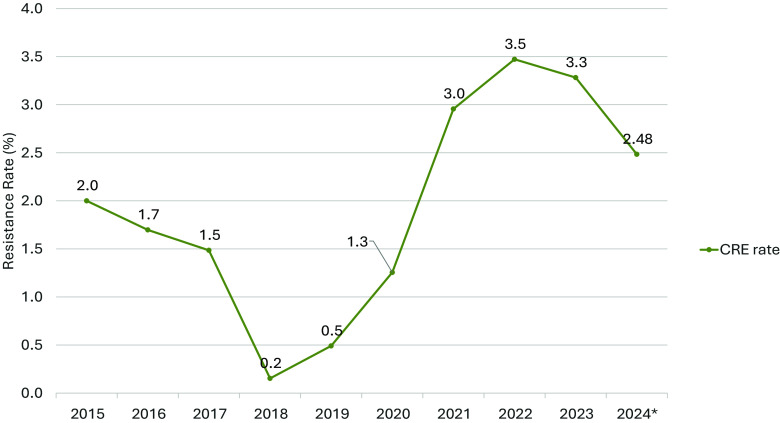
Carbapenem resistance rates by year.

A total of 5165 enterobacterales isolates grew during the study period, of which 104 (%) isolates from 101 patients were found to have carbapenem resistance. A total of 284 CSE isolates from 280 patients were used as comparators. Of the 104 CRE isolates, 9 were isolated in 2015, followed by 14 in 2016, 8 in 2017, 3 in 2018, 4 in 2019, 8 in 2020, 21 in 2021, 15 in 2022, 14 in 2023, and 8 in 2024 (Figure 1). The CRE resistance rate peaked at 3.47 in 2022, a 64% increase from the peak resistance rate before COVID-19 (Figure 2).

### Microbiology and genetic basis of resistance

**Figure 3.**
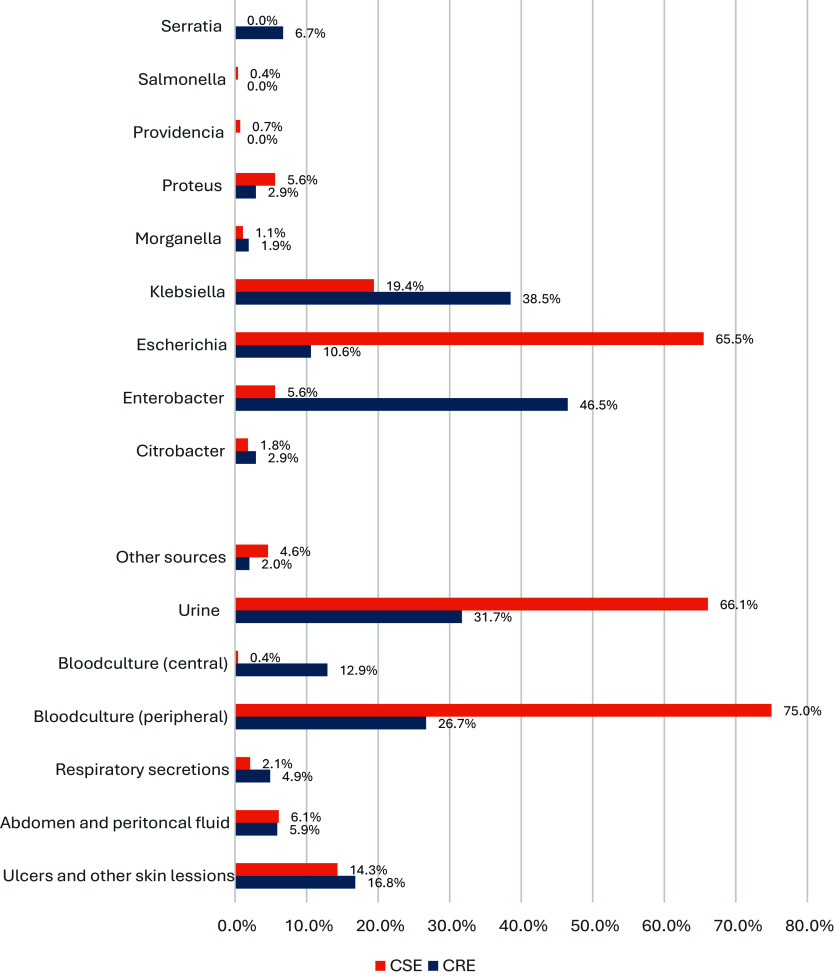
Microbiology and sample source.

CRE cases were more frequently associated with *Enterobacter* (36.5% vs. 5.6%) and *Klebsiella* (38.5% vs. 19.4%), while *Escherichia* was predominantly found in CSE cases (65.5% vs. 10.6% for CRE) (Figure 3, Supplement 1). CRE samples were more frequently obtained from blood cultures, both peripheral (26.7% vs. 7.5%) and central (12.9% vs. 0.4%), compared to CSE. Urine samples were significantly more common in CSE cases (66.1% vs. 31.7% for CRE) (Figure 3, Supplement 1). Of the 104 isolates with carbapenem resistance, 64 (61.5%) had carbapenemase genes identified, 13 (12.5%) indeterminate, and 27 (26%) isolates without carbapenemase detected. Identified genotypes included KPC with 40 cases (38.4%), 21 isolates (20%) with Metallo betalactamase (MBL) and 3 (3%) with OXA production (Figure 1).

### Clinical characteristics and risk factors

Male sex was more common among CRE patients (61.4% vs. 37.1%, *P* < 0.001). CRE patients had a longer length of stay (mean 16.15 ± 17.82 days vs. 2.87 ± 7.74 days for CSE, *P* < 0.001), and were more likely to experience prolonged hospitalization of over 10 days (55.5% vs. 10%, OR 2.42, *P* < 0.001) (Table 1). CRE patients were more often in the ICU (34.7% vs. 3.6%, OR 2.65 [95% CI: 1.9–3.4], *P* < 0.001), while CSE patients were more frequently in walk-in or clinical wards (*P* < 0.001 for both) (Table 1). Among comorbidities, nephropathy (28.7% vs. 13.2%, *P* < 0.001), diabetes mellitus (34.7% vs. 24.3%, *P* = 0.006), and COVID-19 (7.9% vs. 1.4%, *P* = 0.004) were significantly more prevalent in CRE cases (Table 1).

**Table 1.** Risk factors correlated with carbapenem resistance among patients with an Enterobacteria isolate

			Bivariable analysis	
Variable	CRE (n = 101; %)	CSE (n = 280; %)	OR (95% CI)	*P* value
**Patient sex**				
Male	62 (61.4%)	104 (37.1%)	0.99 (0.52–1.46)	<0.001
Age (years)*				
Mean ± SD	56.5 ± 20.9	55.1 ± 19.5		0.059
**Length of stay (days)**	(n = 87)*	(n = 107)*		
Mean ± SD	18.7 ± 17.9	4.6 ± 9		
≥10	56 (64.4%)	17 (15.9%)	2.42 (1.86–2.97)	<0.001
**Comorbidities**				
Asthma	3 (2.9%)	8 (2.9%)	0.04 (–1.3 to 1.4)	1
Peptic ulcer	4 (3.9%)	3 (1.1.%)	1.33 (–0.18 to 2.85)	0.08
COVID-19	8 (7.9%)	4 (1.4%)	1.78 (0.56–3)	0.004
HIV/AIDS	1 (0.9%)	2 (0.8%)	0.33 (–2.08 to 2.74)	1
Cancer (others)	11 (10.9%)	28 (10%)	0.09 (–0.64 to 0.83)	0.85
Lymphoma	2 (1.9%)	3 (1.07%)	0.62 (–1.18 to 2.43)	0.61
Leukemia	3 (2.9%)	2 (0.7%)	1.45 (–0.36 to 3.25)	0.119
Nephropathy	29 (28.7%)	37 (13.2%)	0.97 (0.42 - 1.53)	<0.001
Paralysis	3 (2.9%)	6 (2.1%)	0.33 (–1.07 to 1.74)	0.704
DM	35 (34.7%)	68 (24.3%)	0.5 (0.010–1)	0.05
Connective tissue disorder	2 (1.9%)	7 (2.5%)	–0.24 (–1.83 to 1.35)	0.768
Vasculopathy	65 (64.4%)	145 (51.8%)	0.52 (0.05–1)	0.036
Congestive heart failure	8 (7.9%)	10 (3.6%)	0.84 (–0.12 to 1.8)	0.077
Myocardial infarction	5 (4.9%)	10 (3.6%)	0.34 (–0.76 to 1.44)	0.541
Stroke or TIA	16 (15.8%)	23 (8.2%)	0.74 (0.06–1.43)	0.03
**Antibiotics consumption**				
General use	82 (81.2%)	101 (36.1%)	2.03 (1.48–2.6)	<0.001
Macrolides	4 (3.9 %)	7 (2.5%)	0.475 (–0.78 to 1.73)	0.452
Penicillin derivatives	6 (5.9%)	20 (7.1%)	–0.2 (–1.14 to 0.75)	0.681
Glycopeptides	36 (35.6%)	21 (7.5%)	1.92 (1.32–2.52)	<0.001
Cephalosporins	55 (54.5%)	64 (22.9%)	1.4 (0.92–1.88)	<0.001
Fluoroquinolones	28 (27.7%)	35 (12.5%)	0.99 (0.43–1.55)	<0.001
Carbapenems	41 (40.6%)	12 (4.3%)	2.73 (2.02–3.43)	<0.001
**Invasive procedures**				
General use	60 (59.4%)	103 (36.8%)	0.92 (0.46–1.39)	<0.001
Central venous catheter	37 (36.6%)	15 (5.4%)	2.32 (1.67–2.98)	<0.001
Ventricular drain	2 (1.9%)	7 (2.5%)	–0.24 (–1.83 to 1.35)	0.768
Mechanical ventilation	29 (28.7%)	12 (4.3%)	2.2 (1.48–2.92)	<0.001
Urinary catheter	38 (37.62%)	54 (19.29%)	0.93 (0.43–1.43)	<0.001
Post surgery	33 (32.7%)	58 (20.7%)	0.62 (0.11–1.13)	0.016
Dialysis	20 (19.8%)	14 (5%)	1.55 (0.82–2.27)	<0.001
**Hospital ward**				
Isolation ward	5 (4.9%)	4 (1.4%)	1.27 (–0.07 to 2.6)	0.048
Walk-in	14 (13.9%)	173 (62.5%)	<0.001	
Clinical ward	14 (13.9%)	173 (62.5%)	–2.3 (–2.9–(–1.7))	<0.001
Surgery ward	11 (10.9%)	18 (6.5%)	0.57 (–0.22 to 1.35)	0.156
ICU	35 (34.7%)	10 (3.6%)	2.65 (1.9–3.4)	<0.001
Onco-hematology	5 (4.9%)	8 (2.9%)	0.56 (–0.58 to 1.7)	0.33

Note: All *P*-values are Chi-square except for “age” (*t*-test).

Antibiotic use was strongly associated with CRE, showing an odds ratio of 0.288 (95% CI: 0.148–0.56, *P* < 0.001) (Tables 1-2, Supplement 2-3). Antimicrobial use was more common with CRE (81% vs 36%). Higher rates of glycopeptide (35.6% vs. 7.5%, *P* < 0.001), cephalosporin (54.5% vs. 22.9%, *P* < 0.001), and carbapenem (40.6% vs. 4.3%, *P* < 0.001) use was found in CRE patients (Tables 1-2, Supplement 2). CRE cases involved more frequent use of invasive procedures, such as central venous catheters (36.6% vs. 5.4%, OR 2.32, *P* < 0.001) and mechanical ventilation (28.7% vs. 4.3%, OR 2.2, *P* < 0.001) (Table 1, Supplement 2-3). CRE were also more commonly post-surgical cases (32.7% vs. 20.7%, *P* = 0.016).

**Table 2.** Logistic regression model for potential carbapenem-resistant Enterobacterales risk factors

					Wald test			95% Confidence interval (odds ratio scale)	
Coefficients	Estimate	Standard error	Odds ratio	z	Wald Statistic	df	*p*	Lower bound	Upper bound
(Intercept)	−2.671	0.272	0.069	−9.836	96.739	1	<.001	0.041	0.118
Previous general antibiotic consumption	1.127	0.336	3.088	3.351	11.228	1	<.001	1.597	5.971
Central venous catheter	0.924	0.445	2.520	2.079	4.321	1	0.038	1.054	6.023
Mechanical ventilation	0.164	0.558	1.179	0.295	0.087	1	0.768	0.395	3.516
ICU stay	1.281	0.512	3.601	2.501	6.255	1	0.012	1.319	9.827
Previous carbapenem consumption	1.370	0.436	3.937	3.146	9.895	1	0.002	1.676	9.247
History of COVID-10	1.046	0.821	2.847	1.275	1.625	1	0.202	0.570	14.216
LoS >10 days	1.329	0.350	3.779	3.795	14.404	1	<.001	1.902	7.508

Note. Carbapenem-resistance level “Yes” coded as class 1.

## Discussion

Our study provides insight into the shifting dynamics of carbapenem resistance in the DR over the course of nine years, including years before and after the COVID-19 pandemic. During the pandemic, a marked increase in CRE infections was observed at our facility, with the CRE resistance rate peaking at 3.47% in 2021—a 64% increase compared to pre-pandemic levels in 2015. This rise mirrors trends reported in other parts of Latin America and the United States. One analysis of antimicrobial susceptibility patterns in outpatients across the DR from 2018 to 2021 also revealed an increase in carbapenem resistance for *E. coli* and *Pseudomonas* immediately after the pandemic.^8^ Similarly, reports from the United States (US) also show a rise in AMR during the COVID-19 pandemic.^9^

The widespread overuse of antibiotics in LMICs, such as the DR, is a long-standing challenge that may have been worsened by the pandemic. In the DR, antibiotics are readily accessible without prescriptions in pharmacies and retail stores, and this easy access could contribute to the high prevalence of ESBL-producing organisms.^10^ Aminopenicillins are frequently purchased over the counter, potentially contributing to the high prevalence of ESBL-producing organisms. Previous studies have demonstrated high rates of ceftriaxone resistance, with over 25% of *E. coli* strains resistant in outpatient settings and over 46% of hospitalized adults affected.^8,11,12^ High rates of ESBL-producing bacteria make carbapenems a crucial part of antimicrobial therapy for moderately or severely ill hospitalized patients. The increase in carbapenem resistance observed in our study may reflect the antimicrobial pressure exerted during the pandemic, rising in 2021 and subsequently declining in 2022. COVID-19 infection, prolonged hospital stays, and bloodstream infections were significantly associated with carbapenem-resistant Enterobacteriaceae (CRE) during this period, indicating that critically ill patients are at heightened risk. Therefore, scaling up antimicrobial stewardship programs in the DR and other LMICs is essential to mitigate the spread of CRE.

The predominant carbapenemase genes in our cohort were KPC (57.1%), MBL (37.14%), and OXA (5.7%), underscoring genetic diversity of resistance mechanisms in CRE in the DR.

Some carbapenem-resistant isolates had no detectable carbapenemase genes, potentially due to mechanisms like porin loss or efflux pump overexpression. KPC, first identified in the US in 1996 and in Latin America in 2005, remains the most common carbapenemase in the US.^13^ In contrast, NDM is predominant in the Indian subcontinent and some Balkan nations, while OXA-48 and its variants are prevalent in Europe, the Mediterranean, and North Africa, with sporadic US cases linked to travel.^14^ Understanding the distribution of these genes is essential for selecting optimal antimicrobials to treat carbapenem-resistant infections and guiding hospital formulary decisions.

Our study has several limitations. As a retrospective case-control study, it is subject to selection and information biases due to reliance on existing medical records. Conducted in a single hospital, the findings may not be generalizable or reflect the epidemiology across the DR. The small sample size may affect statistical power and our genotype testing method may vary in sensitivity and specificity. However, it provides a useful clinical and microbiological snapshot of carbapenem resistance over a three-year period, highlighting the impact of the COVID-19 pandemic on AMR patterns. Our study underscores the critical need for ongoing monitoring of AMR, particularly in the context of global health crises such as the COVID-19 pandemic.

## Supporting information

Hernández-Landa et al. supplementary material Hernández-Landa et al. supplementary material

## Supplementary material

For supplementary material accompanying this paper visit http://doi.org/10.1017/ash.2024.503. click here to view supplementary material
