# Supplementary material for: Carbapenem resistance in the Dominican Republic: clinical characteristics, genotypic profiles, and risk factors
Source: Antimicrob Steward Healthc Epidemiol. 2025 Jan 27;5(1):e23. doi: 10.1017/ash.2024.503 (PMC11795434; doi:10.1017/ash.2024.503)
Supplement: Hernández-Landa et al. supplementary material [file S2732494X24005035sup001.docx]

**SUPPLEMENTS**

**Supplement 1.** Microbiology and sample source

|  | **CRE (n = 101)** | **CSE (n = 280)** |
| --- | --- | --- |
| ***Source*** |  |  |
| Ulcers and other skin lessions | 17 (16.8%) | 40 (14.3%) |
| Abdomen and peritoncal fluid | 6 (5.9%) | 17 (6.1%) |
| Respiratory secretions | S (4.9%) | 6 (2.1%) |
| Bloodculture (peripheral) | 27 (26.7%) | 21 (75%) |
| Bloodculture (central) | 13 (12.9%) | 1（0.4%） |
| Urine | 32 (31.7%) | 185 (66.1%) |
| Others | 12 (256) | 13 (4.6%) |
| ***Species*** |  |  |
| Citrobacter | 13 (2.9%) | 5 (1.8%) |
| Enterobacter | 38 (36.5%) | 16 (S.6%) |
| Escherichia | 11 (10.6%) | 186 (65.5%) |
| Klebsiella | 40 (38.5%) | 55 (19.4%) |
| Morganella | 2 (1.9%) | 3 (1.1%) |
| Proteus | 3 (2.9%) | 16 (5.6%) |
| Providencia | 0 | 2 (0.7%) |
| Salmonella | 0 | 1 (0.4%) |
| Serratia | 7 (6.7%) | 0 |

**Supplement 2**. Summary and performance metrics of the Logistic regression model.

|  | | | | | | | | | | | | | | | | | | | | | |
| --- | --- | --- | --- | --- | --- | --- | --- | --- | --- | --- | --- | --- | --- | --- | --- | --- | --- | --- | --- | --- | --- |
| **Model** | | **Deviance** | | **AIC** | | **BIC** | | **df** | | **Χ²** | | **p** | | **McFadden R²** | | **Nagelkerke R²** | | **Tjur R²** | | **Cox & Snell R²** | |
| H₀ |  | 440.677 |  | 442.677 |  | 446.619 |  | 380 |  |  |  |  |  |  |  |  |  |  |  |  |  |
| H₁ |  | 291.005 |  | 307.005 |  | 338.547 |  | 373 |  | 149.672 |  | < .001 |  | 0.340 |  | 0.474 |  | 0.396 |  | 0.325 |  |
|  | | | | | | | | | | | | | | | | | | | | | |

| **Performance metrics** | | | |
| --- | --- | --- | --- |
|  | | **Value** | |
| Accuracy |  | 0.835 |  |
| AUC |  | 0.857 |  |
|  | | | |
